# Scaling, Anisotropy, and Complexity in Near‐Surface Atmospheric Turbulence

**DOI:** 10.1029/2018JD029383

**Published:** 2019-02-08

**Authors:** Ivana Stiperski, Marc Calaf, Mathias W. Rotach

**Affiliations:** ^1^ Department of Atmospheric and Cryospheric Sciences University of Innsbruck Innsbruck Austria; ^2^ Department of Mechanical Engineering University of Utah Salt Lake City UT USA

**Keywords:** anisotropy invariants, complex terrain, similarity theory, turbulent flow

## Abstract

The development of a unified similarity scaling has so far failed over complex surfaces, as scaling studies show large deviations from the empirical formulations developed over flat and horizontally homogeneous terrain as well as large deviations between the different complex terrain data sets. However, a recent study of turbulence anisotropy for flat and horizontally homogeneous terrain has shown that separating the data according to the limiting states of anisotropy (isotropic, two‐component axisymmetric and one‐component turbulence) improves near‐surface scaling. In this paper we explore whether this finding can be extended to turbulence over inclined and horizontally heterogeneous surfaces by examining near‐surface scaling for 12 different data sets obtained over terrain ranging from flat to mountainous. Although these data sets show large deviations in scaling when all anisotropy types are examined together, the separation according to the limiting states of anisotropy significantly improves the collapse of data onto common scaling relations, indicating the possibility of a unified framework for turbulence scaling. A measure of turbulence complexity is developed, and the causes for the breakdown of scaling and the physical mechanisms behind the turbulence complexity encountered over complex terrain are identified and shown to be related to the distance to the isotropic state, prevalence of directional shear with height in mountainous terrain, and the deviations from isotropy in the inertial subrange.

## Key Points

- A pathway toward a unified theory of near‐surface atmospheric turbulence is provided
- The separation according to the limiting states of anisotropy significantly improves the collapse of data onto common scaling relations
- Physical processes responsible for turbulence complexity are identified

## 1 Introduction

Atmospheric surface layer (ASL) similarity theory was developed as a unified theory of statistically stationary turbulence over horizontally homogeneous and flat terrain in what can be considered a canonical surface layer (e.g., Monin & Yaglom, 1971). Although never meant to be employed over heterogeneous and nonflat surfaces, the lack of a better framework has thus far led to similarity theory being employed in weather prediction and climate models over all types of terrain (cf. Rotach et al., 2017). Given the prevalence of heterogeneity of the Earth land surface (e.g., Rotach et al., 2014), adaptations were developed by reconciling theory and application under the principle of local homogeneity in order to be able to model real flows over heterogeneous surfaces. Meaning that over small enough regions, sampled long enough, what a priori might resemble a heterogeneous surface can ultimately be interpreted as homogeneous under a moving‐equlibrium hypothesis (Yaglom, 1979). While these practical adjustments work well for regions with weak heterogeneities (e.g., Sfyri et al., 2018), similarity relationships become severely challenged in complex terrain (e.g., Martins et al., 2009; Nadeau et al., 2013; Sfyri et al., 2018) where topographic complexity, land use characteristics, complexity of the flow, and their interactions cause turbulence to increasingly depart from canonical surface layer structure (e.g., Grachev et al., 2016; Nadeau et al., 2013; Oldroyd et al., 2016; Stiperski & Rotach, 2016). In this work, reference to complex terrain is understood as topographic perturbations that induce spatial and/or temporal perturbations to the atmospheric flow with a timescale shorter than that of the diurnal cycle or mesoscale phenomena (e.g., sloped terrain, ground roughness and thermal patchiness, and obstacles). The flow complexity, on the other hand, refers to phenomena such as low‐level jets, flow separation, and upside‐down boundary layers, known to also lack a canonical surface layer structure.

Numerous studies have illustrated the adequacy of similarity theory under idealized terrain and flow conditions (e.g., Panofsky & Dutton, 1984; Wyngaard, 2010). Nonetheless, an important degree of scatter still exists, particularly for horizontal velocity variances, commonly assumed not to obey surface layer scaling (e.g., Banerjee et al., 2015; Chamecki et al., 2017; Kaimal & Finnigan, 1994; Wyngaard, 2010). This scatter also persists despite the advanced postprocessing techniques and progressively more restrictive quality criteria imposed on the data. In an effort to overcome these challenges, Stiperski and Calaf (2018) employed a novel approach by examining traditional similarity scaling relations over flat and horizontally homogeneous terrain based on clustering the data according to anisotropy. Results of this work illustrated a strong dependence between the quality of the scaling fit and the characteristic topology of the turbulent flow, showing that the similarity scaling significantly improves when the turbulent flow is a priori classified according to the anisotropy type. In essence, results illustrated that isotropic and two‐component axisymmetric‐type turbulence scale the best (i.e., show closest collapse on a scaling line), although for horizontal velocity components, the two types of anisotropy were shown to follow different scaling curves. This finding could explain the commonly encountered large scatter observed for scaled standard deviations of horizontal velocities. On the other hand, one‐component turbulence strongly departs from common scaling curves. These results, together with the possibility of predicting the anisotropy type based on larger‐scale variables as shown in Stiperski and Calaf (2018), promised to be a powerful tool to improve similarity scaling relations.

In complex terrain, on the other hand, despite the progressively more severe restrictions imposed on the experimental data (cf. Stiperski & Rotach, 2016), significant scatter and a relevant degree of discrepancy between the experimental data and similarity relationships are more evident. Even more, all the studies examining the applicability of surface layer scaling for data obtained over diverse complex settings (e.g., Babić, Rotach, & Klaić, 2016, Babić, Večenaj, & De Wekker, 2016; de Franceschi et al., 2009; Grachev et al., 2016; Kral et al., 2014; Martins et al., 2009; Nadeau et al., 2013; Park & Park, 2006; Sfyri et al., 2018) show that the scaling relations differ not only from the functional relations obtained over flat and horizontally homogeneous terrain but also from site to site, suggesting that scaling might be inherently local (i.e., location dependent) and therefore negating the possibility of a unified theory of turbulence, (i.e., applicable over all types of surfaces). The search for an additional scaling variable in complex terrain that could explain these discrepancies has so far been proven unsuccessful, as the only systematic study to date (Sfyri et al., 2018) found no clear relationship between scaling and slope angle, at least for the scaled standard deviations of scalars. Still, the data from progressively more complex surfaces do show larger deviations from the scaling curves and generally larger scatter, even if the exact mechanism behind this finding escapes clear explanation.

In this work, and based on the earlier approach first introduced in Stiperski and Calaf (2018), we present a new interpretation of the a priori mismatch of near‐surface data in complex terrain and traditional scaling relations based on the anisotropy of the turbulence stress tensor. The results show that similar to flat and horizontally homogeneous terrain, separating the complex terrain data according to anisotropy significantly improves scaling, offering a pathway toward a unified theory of turbulence. In addition, we provide a novel approach that defines complexity as a departure of turbulence structure from its canonical form. We quantify this departure through deviations of the data from the scaling curves obtained over flat and horizontally homogeneous terrain. Thus, complexity is not only exclusively associated with terrain characteristics, but also to the actual resultant turbulence structure. The physical mechanisms causing this complexity are then identified. This new definition of complexity facilitates comparison between data sets collected in regions with different atmospheric and topographic characteristics.

The paper is organized as follows: in section 2 the data sets and postprocessing methods are presented, the anisotropy analysis is reviewed, and scaling relations introduced; section 3 presents the relationship between similarity scaling and the anisotropy of turbulence over complex terrain; section 4 identifies a measure of turbulence complexity and examines its relation to the physical mechanisms acting in complex terrain; an extended discussion of the results and implications for similarity theory as well as conclusions are provided in section 5.

## 2 Methodology

### 2.1 Data Sets

In this study we examine turbulence measurements from 12 flux towers located on surfaces of different complexity, ranging from flat to highly complex mountainous terrain and over different types of surface. These are part of well‐known data sets and include the tower at Cabauw experimental site for atmospheric research (Cesar) of the Royal Netherlands Meteorological Institute (e.g., Beljaars & Bosveld, 1997), the Cooperative Atmosphere‐Surface Exchange Study 1999 (CASES‐99; Poulos et al., 2002), the Terrain‐induced Rotor Experiment (T‐Rex; Grubišič et al., 2008), the Mountain Terrain Atmospheric Modeling and Observations (MATERHORN; Fernando et al., 2015), the Second Meteor Crater Experiment (METCRAX II; Lehner et al., 2016), and the Innsbruck Box (i‐Box; Rotach et al., 2017). A detailed description of the data sets is given in Table 1 .

**Table 1:** Information on the Data Sets Used in the Study

Station	Official name	Short name	Location	Terrain complexity	Measurements heights (m)	Slope angle (°)	Surface type	Data length
**CASES‐99**	CASES‐99	CA	Kansas	Flat	5, 10, 20, 30, 40, 50, 55	< 0.5	Grassland	October 1999
**Cabauw**	Cabauw	CB	Netherlands	Flat	3, 60	< 0.5	Grassland	July‐October 2007
**T‐RexC**	Central Tower	TRC	California	Flat valley floor	5, 10, 15, 20, 25, 30	< 0.5	Desert	March‐May 2006
**i‐Box0**	CS‐VF0	iB0	Austria	Flat valley floor	4, 8.7, 16.9	< 0.5	Mixed Agricultural	January‐December 2015
**i‐Box1**	CS‐SF1	iB1	Austria	Flat foothills	6.6	1	Alpine meadow and agricultural	January‐December 2015
**METCRAX II**	Near Tower	MC	Arizona	Gentle Slope	5, 10, 15, 20, 25, 30, 35, 40, 45, 50	1	Desert	October 2013
**T‐RexW**	West Tower	TRW	California	Gentle Slope	5, 10, 15, 20, 25, 30	3.25	Desert	March‐May 2006
**MATERHORN ES4**	ES4 Tower	MT4	Utah	Gentle Slope	0.47, 2.05, 5.12, 10, 20, 26.5	4	Desert	September‐October 2013
**MATERHORN ES5**	ES5 Tower	MT5	Utah	Gentle Slope	0.55, 2.14, 5.13, 10.13, 20.08	6.4	Desert	September‐October 2013
**i‐Box10**	CS‐NF10	iB10	Austria	Steep Slope	6.2	10	Alpine meadow	January‐December 2015
**i‐Box27**	CS‐NF27	iB27	Austria	Steep Slope	6.8	27	Alpine meadow	January‐December 2015
**i‐BoxTop**	CS‐MT21	iBTop	Austria	Mountain Top	4.67	21	High‐Alpine Vegetation	April‐October 2015

The data set that conforms to the flat and horizontally homogeneous terrain the best is CASES‐99. Already studied in Stiperski and Calaf (2018), it forms the basis of the current analysis. The data consist of a month of measurements from a 60‐m tower with 7 levels of sonic anemometers. Due to issues with the anisotropy of the CSAT3 measurements during stable periods identified in Stiperski and Calaf (2018), here we only study the levels with ATI‐K probes in stable conditions. Cabauw data can also be considered flat, however, horizontally weakly inhomogeneous (Sfyri et al., 2018). The other data sets were chosen according to their increasing terrain complexity. Although located at the almost flat valley floor, the Central tower from T‐Rex shows systematic deviations from flat terrain scaling (cf. Babić, Večenaj, & De Wekker, 2016), caused by its setting within a mountain valley. The i‐Box0 valley floor site (see Table 1), apart from being located in a narrower valley than T‐Rex, is additionally characterized by larger surface heterogeneity, given that it is surrounded by mixed agricultural land. The rest of the data sets are located on slopes of various steepness and are strongly influenced by flows associated with sloped terrain (e.g., thermally driven katabatic and anabatic flows and dynamically driven wind systems) and/or heterogeneity. The i‐Box1 station has a small slope angle, however, the influence of surface heterogeneity (corn and meadows) for this station is larger than the influence of sloping terrain because the dominant wind direction is across the slope. The same is true for T‐Rex West tower, although there the wind rose also shows a large influence of katabatic flows as well as downslope windstorms (Babić, Večenaj, & De Wekker, 2016). On the other hand, deep katabatic flows with jet maxima between 20 and 40 m above ground level (agl) develop regularly at the METCRAX II NEAR tower (cf. Lehner et al., 2016; Savage et al., 2008). Persistent shallow katabatic flows with a jet maximum at around 5 m agl are also found at MATERHORN ES4 and ES5 towers located at the top of a relatively shallow slope below a couloir (Grachev et al., 2016). Even shallower katabatic flows develop at the steeper i‐Box10 and i‐Box27 stations. The i‐Box mountain top station (i‐BoxTop) represents the most complex site due to its location on a ridge exposed to flow from all sides that, depending on wind direction, responds to very different slope angles. Because operating continuous turbulence measurements at this station is challenging, only 7 months of measurements were analyzed in this study, as opposed to the other i‐Box stations where 1 year of data was analyzed.

The data sets (in both Table 1 and future figures) are a priori subjectively ordered according to their slope angle, with cooler colors representing gentler slopes (flat terrain being considered more ideal than the flat valley floor locations) and warmer colors progressively steeper slope angles (4–27°).

### 2.2 Data Treatment and Quality Control

**Figure 1:**
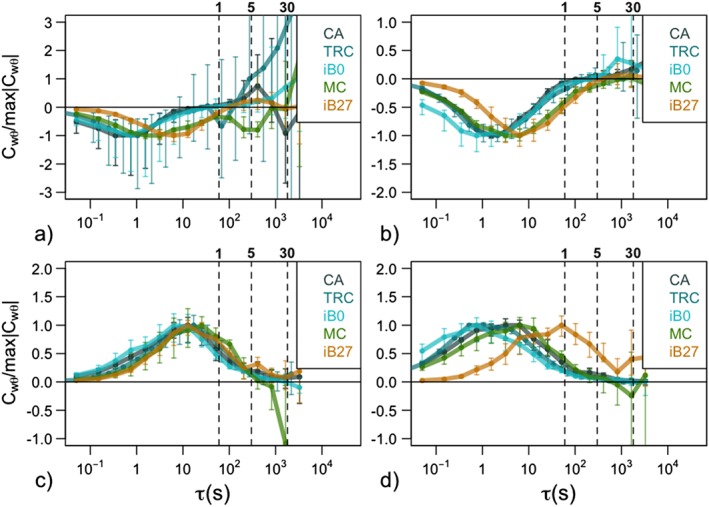
Multiresolution flux decomposition of heat flux for the example nights with (a) strongly stable, (b) weakly stable, (c) strongly unstable, and (d) weakly unstable stratification, respectively, for the lowest measurement level of different data sets (shown in color, see Table 1 for abbreviations). The colored lines represent medians and are normalized by their maximum value within the turbulence scales for each data set so as to eliminate differences in magnitude. Error bars represent the 25th and 75th percentiles. Vertical dashed lines indicate timescales of 1, 5, and 30 min, respectively.

To remove the inconsistencies in data processing applied by different groups responsible for each of the data sets, we reanalyzed all the data with a processing routine described in Stiperski and Calaf (2018). First, the multiresolution flux decomposition (MRD; e.g., Vickers & Mahrt, 2003) technique was used to determine the optimal averaging time for daytime and nighttime turbulence (Figure 1). As in Stiperski and Calaf (2018) we separate the data into strongly and weakly stable/unstable regimes to examine how the averaging time depends on the stability. The results show that for the examined data sets a 1‐min averaging time for stable stratification and 30‐min averaging time for unstable stratification generally capture the majority of turbulence contributions to the flux while eliminating most of the (sub)mesoscale effects. The obvious exception here is the i‐Box27 steep slope station (orange line in Figure 1). At that station very stable conditions are rarely encountered; therefore, 1‐min average slightly underestimates the nighttime fluxes. The data were then detrended and block averaged to the given averaging time. Double rotation was used to align the coordinates into the streamwise coordinate system. Zero‐plane displacement information was applied to the stations where this information was available. For i‐Box measurements, the zero‐plane displacement was calculated based on the measurements of surrounding vegetation height (Sfyri et al., 2018). For the two T‐Rex towers the values from the study of Babić, Večenaj, and De Wekker (2016) were used. For the other data sets the zero‐plane displacement was assumed to be zero given the generally low vegetation height.

Mean wind speed and air temperature gradients needed for calculating the gradient Richardson number *R*_*i*_ were determined for data sets with multiple measurement levels (i.e., CASES‐99, T‐Rex, METCRAX II, MATERHORN, and i‐Box0). In order to determine the local wind speed gradient at each measurement height, analytic profiles were fit through the entire tower length. Different analytic formulations were needed for each data set due to profiles having different characteristics, particularly in case of the existence of low‐level jets. The formulations used were $x=a+b\;z+c\;z^{2}+d\;\log(z)$ for CASES‐99, $x=a+b\;z+c\;z^{2}+d\;\log(z)+e\;\log{\left(z\right)}^{2}$ for T‐Rex, $x=a+b\;z+c\;z^{2}+d\;z^{3}+e\;\log(z)+f\;\log{\left(z\right)}^{2}$ for METCRAX II, $x=a+b\;\log(z)+c\;\log{\left(z\right)}^{2}$ for MATERHORN, and finally $x=a+b\;\log(z)+c\;\log{\left(z\right)}^{2}+d\;\log{\left(z\right)}^{3}$ for i‐Box0. As a quality check, only those wind speed gradients in which the root‐mean‐square error of the best fit was lower than 0.3 m/s were taken into account.

All turbulence data were required to pass the basic quality control (test of physical limits) as well as to satisfy the stationarity test given by Foken and Wichura (1996) at its standard 30% level. As in Stiperski and Calaf (2018), the stationarity criterion was dropped for small fluxes: that is, for very unstable conditions stationarity of the momentum flux was not required, while for near‐neutral conditions the same was true for the stationarity of the heat flux. For data sets with multiple levels, the requirement that the gradient Richardson number be smaller than 0.25 was also imposed (cf. Grachev et al., 2013). As shown in Stiperski and Calaf (2018), existence of unstably stratified turbulence during nighttime points to nonlocal sources of turbulence and cannot be expected to follow scaling. In order to filter these counter gradient fluxes, theoretical incoming short‐wave radiation was used to determine sunrise and sunset times together with the conservative cross‐over time of the daily cycle of sensible heat flux. This was particularly important for i‐Box stations where a year of data was analyzed, meaning that sunset and sunrise times varied significantly. No flux corrections were applied to the data, the same as in Stiperski and Calaf (2018).

### 2.3 Anisotropy

Traditionally, the Reynolds stresses ( $\bar{u_{i}^{\prime }u_{j}^{\prime }}$) can be decomposed into an isotropic and anisotropic contribution, the latter being the only one contributing to the transport of momentum (Pope, 2000). The anisotropy contribution to scalar fluxes is also assumed to be important. The sum of the isotropic components of the Reynolds stress tensor is traditionally referred to as twice the turbulent kinetic energy ( $2e=\bar{u_{i}^{\prime }u_{i}^{\prime }}$). In the above notation, a prime indicates a departure from a time‐averaged quantity, and the overbar indicates the time‐averaging operation. Additionally, the indices *i*,*j* vary between 1 and 3, in reference to the traditional Cartesian coordinate reference system with 1 indicating the streamwise, 2 the spanwise, and 3 the surface‐normal directions, respectively. The results are, however, independent of the coordinate system used, as long as it is orthogonal.

The deviatoric anisotropy stress tensor defined as,

(1) $$a_{ij}\equiv \bar{u_{i}^{\prime }u_{j}^{\prime }}-\frac{2}{3}e\delta_{ij},$$

and in nondimensional form (normalized by 2*e*) as,

(2) $$b_{ij}=\frac{\bar{u_{i}^{\prime }u_{j}^{\prime }}}{\bar{u_{l}^{\prime }u_{l}^{\prime }}}-\frac{1}{3}\delta_{ij},$$

has long been studied in relationship to, for example, the pressure‐strain correlation to develop models that capture the return‐to‐isotropy process once mean velocity gradients stop acting on the flow (Choi & Lumley, 2001; Lumley, 1979; Lumley & Newman, 1977; Sarkar & Speziale, 1990; Rotta, 1951). Based on Lumley's work (Lumley, 1979; Lumley & Newman, 1977), it is possible to reduce the original three‐dimensional problem characterized by six independent terms (normalized deviatoric anisotropy stress tensor) into a simpler problem with two degrees of freedom, based on the anisotropy invariants, *η* and *ξ* (Pope, 2000) that are also functions of the eigenvalues (*λ*_*i*_, *i* = 1,2,3) of the anisotropy stress tensor. The first invariant (*η*) is positive definite and provides a measure of the degree of anisotropy in the flow field (large values indicating intense anisotropy and small values indicating near‐isotropic behavior). The second invariant (*ξ*) can be positive or negative, indicating that the flow is dominated by one‐component turbulence when positive, and by two‐component turbulence when negative. These invariants can be mathematically determined from the normalized deviatoric anisotropy stress tensor as (Pope, 2000)

(3) $$6\eta^{2}=b_{ij}b_{ji}\;\text{and}\;6\xi^{3}=b_{ij}b_{jk}b_{ki}.$$

**Figure 2:**
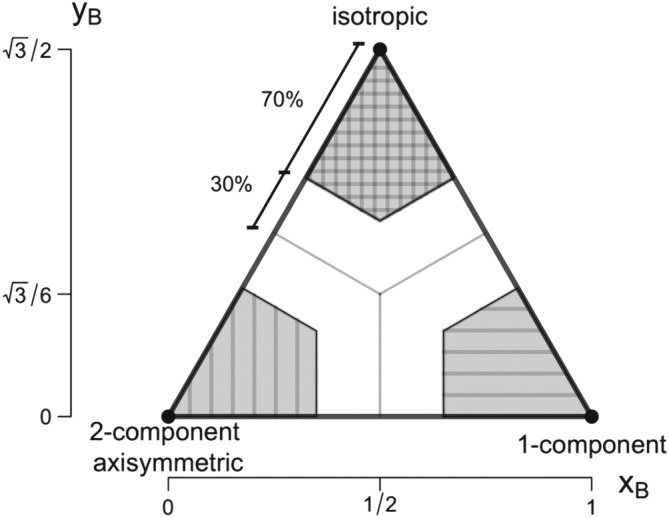
Barycentric Lumley Triangle as a function of the anisotropy stress tensor eigenvalues and represented through the linearized coordinates x_B_ and y_B_. The shading indicates regions of the triangle that were selected as pure limiting states of anisotropy.

As a result, it is possible to represent any realizable state of turbulence on a single two‐dimensional nonlinear map, known as the Lumley Triangle (LT; Lumley, 1979; Pope, 2000). Here, instead, we use a modification of the original LT, the Barycentric Lumley Triangle (BLT; Banerjee et al., 2007, see Figure 2 and Table 2), that overcomes the complexity associated with the nonlinearity of the LT by equally weighing the different limiting states of turbulence anisotropy. The corresponding coordinates (*x*_*B*_, *y*_*B*_) of this linearized 2‐D map are related to the eigenvalues as

(4) $$x_{B}=C_{1c}x_{1c}+C_{2c}x_{2c}+C_{3c}x_{3c}=C_{1c}+C_{3c}\frac{1}{2},$$

(5) $$y_{B}=C_{1c}y_{1c}+C_{2c}y_{2c}+C_{3c}y_{3c}=C_{3c}\frac{\sqrt{3}}{2},$$

with the corresponding weights (*C*_*ic*_) written as *C*_1*c*_ = λ_1_ − λ_2_, *C*_2*c*_ = 2(λ_2_ − λ_3_), and *C*_3*c*_ = 3*λ*_3_ + 1, with *x*_1*C*_ = (1,0), *x*_2*C*_ = (0,0), and $x_{3C}=(1/2,\sqrt{3}/2)$ indicating the limiting states of turbulence anisotropy in the BLT. Both invariant maps are equivalent given the existing relationship between the anisotropy invariants (*η* and *ξ*) and the eigenvalues of the normalized anisotropy tensor (λ_*i*_; Spencer, 1971),

(6) $$\eta^{2}=\frac{1}{3}(\lambda_{1}^{2}+\lambda_{1}\lambda_{2}+\lambda_{2}^{2})$$

(7) $$\xi^{3}=-\frac{1}{2}\lambda_{1}\lambda_{2}(\lambda_{1}+\lambda_{2}).$$

**Table 2:** Summary of the Special States of the Reynolds Stress Tensor in Terms of the Invariants (η , ξ), and the Eigenvalues of the Anisotropy Stress Tensor as Described by the Lumley Triangle

Cases	Invariants	Eigenvalues	Shape ellipsoid
Isotropic	*η* = *ξ* = 0	λ_1_ = λ_2_ = λ_3_ = 0	Sphere
Two‐component axisymmetric	$\eta =\frac{1}{6},\xi =-\frac{1}{6}$	$\lambda_{1}=\lambda_{2}=\frac{1}{6}$	Disk
One‐component	$\eta =\xi =\frac{1}{3}$	$\lambda_{1}=\frac{2}{3},\lambda_{2}=\lambda_{3}=-\frac{1}{3}$	Line
Axisymmetric, one large eigenvalue	*η* = *ξ*	$-\frac{1}{3}\leq \lambda_{1}=\lambda_{2}\leq 0$	Prolate spheroid
Axisymmetric, one small eigenvalue	*η* = −*ξ*	$0\leq \lambda_{1}=\lambda_{2}\leq \frac{1}{6}$	Oblate spheroid
Two‐component	$\eta ={\left(\frac{1}{27}+2\xi^{3}\right)}^{1/2}$	$\lambda_{1}+\lambda_{2}=\frac{1}{3}$	Ellipse

*Note.* The fourth column introduces the corresponding ellipsoid shape described by the eigenvectors (Pope, 2000).

Stiperski and Calaf (2018) have shown that over flat terrain the limiting states of anisotropy can be associated with the dominance of different terms in the turbulent kinetic energy balance. Under unstable stratification isotropic turbulence occurs when buoyancy production dominates shear production of turbulence and two‐component turbulence when the opposite is true. Under stable stratification, however, isotropic turbulence is shear driven, while one‐component turbulence occurs when turbulence kinetic energy (TKE) production is nonlocal and buoyancy damping is larger than shear production.

Finally, it is important to reiterate that the shape associated with the limiting states of anisotropy refers to the representation in eigenvalue space and not to the physical shape of turbulence (Simonsen & Krogstad, 2005). More detail on the analysis of turbulence anisotropy can be found in Stiperski and Calaf (2018).

### 2.4 Scaling

Following Stiperski and Calaf (2018) we examine the influence of turbulence anisotropy on near‐surface similarity within the local scaling framework (cf. Nieuwstadt, 1984a, 1984b). Turbulent quantities are therefore scaled with the fluxes obtained at the corresponding measurement height *z*. The local Obukhov length Λ is defined as $\Lambda =\frac{-u_{*}^{3}\theta_{v}}{\kappa g\bar{w^{\prime }\theta^{\prime }}}$, where *θ*_*v*_ is the mean virtual potential temperature, *κ* the von Karman constant, approximately equal to 0.4, *u*_∗_ the local friction velocity computed as $u_{*}={\left({\bar{u^{\prime }w^{\prime }}}^{2}+{\bar{v^{\prime }w^{\prime }}}^{2}\right)}^{\frac{1}{4}}$ and $\bar{w^{\prime }\theta^{\prime }}$ is the local heat flux. The quantity (*z* − *d*)/Λ, where *d* is the displacement height, represents the local stability. We also define the local temperature scale as $\theta_{*}=-\frac{\bar{w^{\prime }\theta^{\prime }}}{u_{*}}$. The following functional forms of the surface layer flux‐variance similarity relationships are used for reference: for the standard deviations of velocity components (Φ_*u*_,Φ_*v*_,Φ_*w*_), following Panofsky and Dutton (1984),

(8) $$\Phi_{w}=\frac{\sigma_{w}}{u_{*}}=\left\{\begin{array}{c} 1.25{\left(1+3\frac{z}{\Lambda }\right)}^{\frac{1}{3}}\text{for}\frac{z}{\Lambda }>0\\ 1.25{\left(1-3\frac{z}{\Lambda }\right)}^{\frac{1}{3}}\text{for}\frac{z}{\Lambda }<0 \end{array}\right.$$

(9) $$\Phi_{u,v}=\frac{\sigma_{u,v}}{u_{*}}=\left\{\begin{array}{c} 2.55{\left(1+3\frac{z}{\Lambda }\right)}^{\frac{1}{3}}\text{for}\frac{z}{\Lambda }>0\\ 2.55{\left(1-3\frac{z}{\Lambda }\right)}^{\frac{1}{3}}\text{for}\frac{z}{\Lambda }<0 \end{array}\right.$$

for temperature standard deviation (Φ_*θ*_), taking the reference curve from Sfyri et al. (2018),

(10) $$\Phi_{\theta }=\frac{\sigma_{\theta }}{\theta_{*}}=\left\{\begin{array}{c} 2+6.7\cdot 10^{-4}{\frac{z}{\Lambda }}^{-1.42}\text{for}\frac{z}{\Lambda }>0\\ 1.67-0.016{\left(\frac{z}{\Lambda }\right)}^{1}\text{for}-0.05<\frac{z}{\Lambda }<0\\ 1.95{\left(0.05-\frac{z}{\Lambda }\right)}^{\frac{1}{3}}\text{for}\frac{z}{\Lambda }<-0.05 \end{array}\right.$$

and for turbulence dissipation rate (Φ_*ε*_), following Thiermann (1990),

(11) $$\Phi_{\varepsilon }=\frac{kz\varepsilon }{u_{*}^{3}}=\left\{\begin{array}{c} {\left(1+4\frac{z}{\Lambda }+16{\left(\frac{z}{\Lambda }\right)}^{2}\right)}^{\frac{1}{2}}\text{for}\frac{z}{\Lambda }>0\\ {\left(1-3\frac{z}{\Lambda }\right)}^{-1}-\frac{z}{\Lambda }\text{for}\frac{z}{\Lambda }<0 \end{array}\right.$$

In the stable z‐less limit, Sorbjan (1987) suggested the following constant values of the flux variance relationships:

(12) $$\Phi_{w}=1.6,\;\Phi_{u,v}=3.1.$$

A number of measures are used to quantify the amount of scatter between the data sets and the agreement with the reference scaling curves. These quantitative measures refer to the following different types of scatter:

1. Scatter of the data within a data set. For this purpose we calculate the interquartile range,
  (13) $$\delta =Q_{3}(y)-Q_{1}(y).$$
  Here *Q*_1_ and *Q*_3_ are the 25th and 75th percentiles, and *y* are the scaled data corresponding to the different variables, *y* = *u*,*v*,*w*,*θ*,*ε*.
2. Deviation of the scaled data from the reference curve for a given data set is quantified by calculating the absolute deviations
  (14) $$\Delta \Phi_{y}=|y-\Phi_{y}|,$$
  where Φ_*y*_ are the respective scaling curves (equations (8), (9), (10), (11)). For stable conditions the z‐less limit (equation (12)) is used for the velocity components. For data separated according to anisotropy, the distance to the closest scaling line is employed. This implies that for standard deviations of horizontal velocity components under isotropic conditions, the reference curve used in the calculation is Φ_*w*_, and not Φ_*u*,*v*_ (cf. Stiperski & Calaf, 2018). The median (*M*) of the absolute deviations,
  (15) $$\bar{\Delta \Phi_{y}}=M(\Delta \Phi_{y}),$$
  is used to quantify the mean disagreement of each data set with the respective scaling, and therefore to quantify the complexity of the data set.
3. Degree to which different data sets agree with each other and/or the reference curve. While the value of $\bar{\Delta \Phi_{y}}$ for each data set already provides an indicator of the improvement of scaling, it is sensitive to the choice of the scaling curve. An inadequate choice might lead to large values of $\bar{\Delta \Phi_{y}}$, even if those values were consistent among the different data sets. Therefore, as a better indicator of the improvement in scaling, that is, decreased variability in $\bar{\Delta \Phi_{y}}$ between the different data sets, we use the interquartile range,
  (16) $$IQR=Q_{3}(\bar{\Delta \Phi_{y}})-Q_{1}(\bar{\Delta \Phi_{y}}).$$
  It is calculated from the 12 $\bar{\Delta \Phi_{y}}$s for each scaling variable and stability. This measure provides information on the discrepancies between the different data sets and the scaling curves and in that sense quantifies how *location dependent* the scaling for different data sets is, and how much improvement is brought about by the new approach of Stiperski and Calaf (2018).

## 3 Results

**Figure 3:**
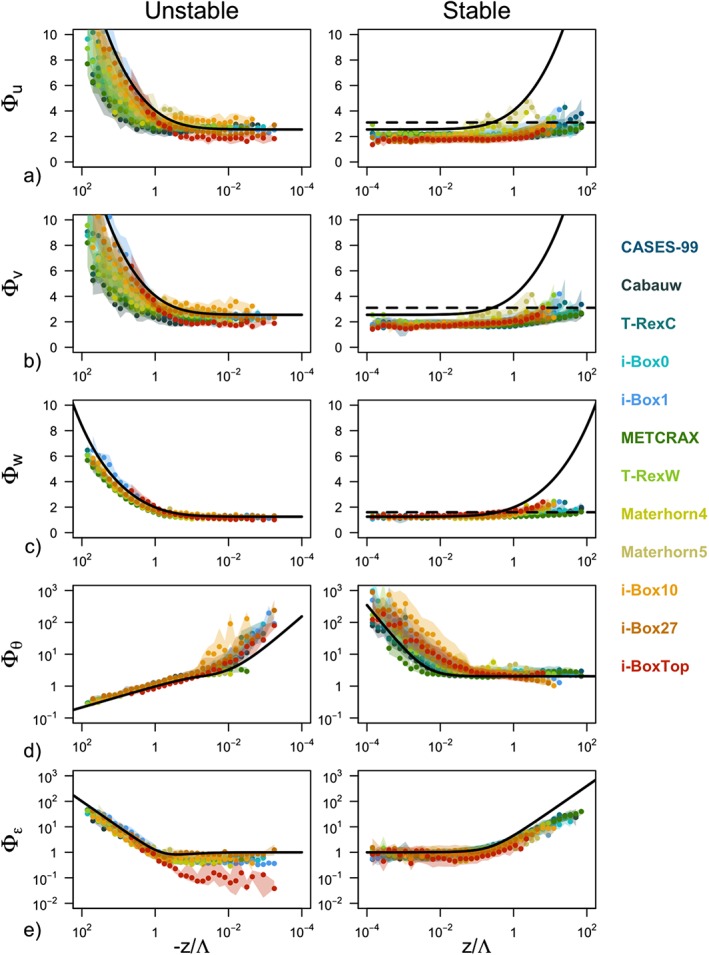
Scaling relations of the standard deviation of (a) streamwise velocity (Φ_u_), (b) spanwise velocity (Φ_v_), (c) surface‐normal velocity (Φ_w_), (d) temperature (Φ_θ_), and (e) TKE dissipation rate (Φ_ε_) as a function of the local stability z/Λ for unstable (left) and stable (right) stratification. Colors represent different data sets described in Table 1. Points represent medians calculated over the bins of logarithmically spaced z/Λ, while the shading corresponds to the interquartile range. The full black lines correspond to the traditional scaling relations (equations (8), (9), (10), (11)) and dashed lines to the z‐less scaling for each variable (equation (12)).

**Figure 4:**
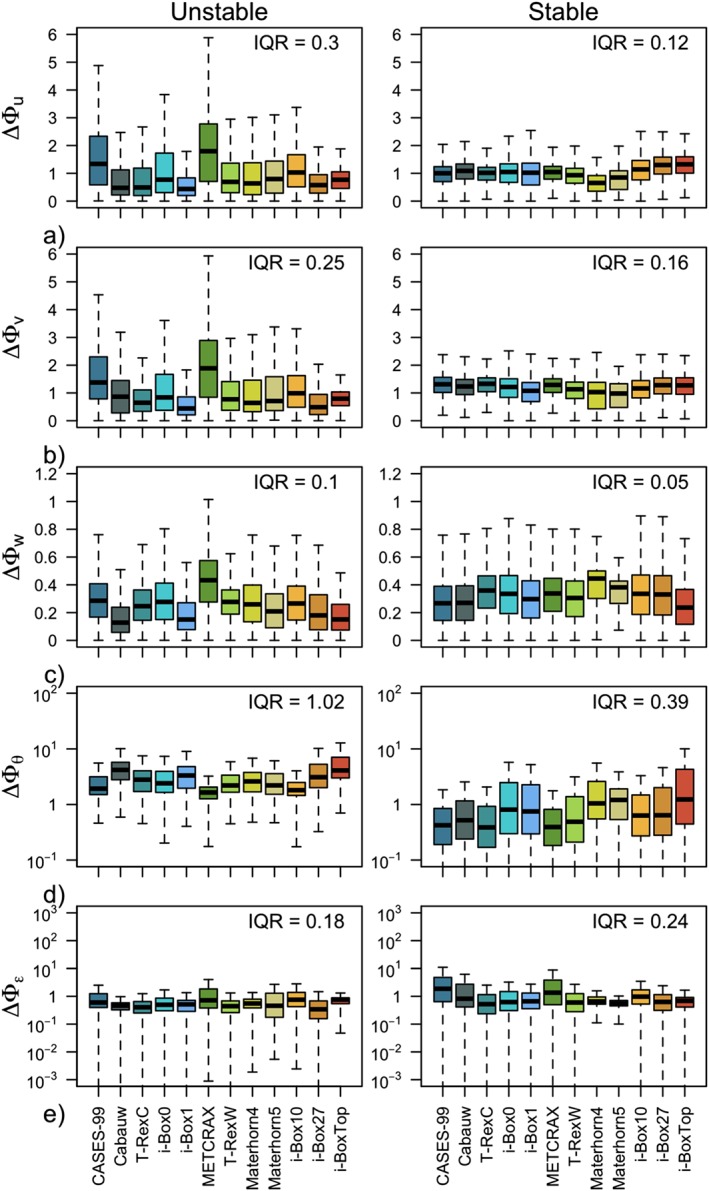
Box plots of absolute deviations (a)–(e) ΔΦ_y_ where y = u,v,w,θ, and ε, respectively, of the scaled data from the corresponding scaling relations (equations (8), (9), (10), (11), (12)): as a function of the data set (color), for unstable (left column) and stable (right column) stratification. Note the different vertical axes for each variable. The value of the interquartile range (I Q R) is listed in each panel.

The scaled standard deviations of high‐quality stationary data for each of the 12 data sets are shown in Figure 3 in comparison with the traditional similarity relations from horizontally homogeneous and flat terrain (equations (8), (9), (10), (11), (12)). For visualization purposes the data from each data set are binned and the median of each bin is displayed. The spread of the data is shown as the shading and corresponds to an interquartile range of each bin. The absolute deviations between the data and the reference scaling curves as well as interdataset spread are shown in Figure 4 .

The results show large scatter both within each individual data set (large shaded area in Figure 3 corresponding to *δ*) as well as between the different data sets (large *IQR* values in Figure 4, i.e., variability of $\bar{\Delta \Phi_{y}}$ between the data sets is on the order of 30%), confirming the location‐dependent nature of scaling in complex terrain. The large scatter within each data set is particularly clear for horizontal velocity components Φ_*u*,*v*_ in the very unstable region (cf. Figures 3a and 3b), to the point that the similarity relations can be deemed meaningless in that case. The same is true for the near‐neutral regions for scaled temperature Φ_*θ*_ and less so for the scaled TKE dissipation rate Φ_*ε*_. Notably, the vertical velocity variance exhibits good scaling behavior throughout, regardless of the data set. This is particularly interesting given the large disparity of data sets used in this work, representative of very different terrain and flow complexities as well as vertical coordinates, which over flat terrain represent the vertical and in complex terrain the slope‐normal direction. In the stable regime in general, the data scatter suggests a better collapse to a scaling curve (smaller *IQR* values in Figure 4 than for unstable data), particularly for weakly stable conditions of the velocity components Φ_*u*,*v*,*w*_. It is interesting to note, however, that the scaled standard deviation of streamwise velocity Φ_*u*_ seems to suggest an ordering according to the data sets in the near‐neutral regime, with some data sets showing a higher neutral limit than others, but all exhibiting a value lower than that established for horizontally homogeneous and flat terrain. The deviations from the reference scaling curves (equations (8) and (9)) in the very stable regime are quite substantial, indicative of, though not confirming, z‐less scaling. This general behavior is slightly different for Φ_*θ*_, where large scatter, mirroring that of the very unstable region of horizontal velocities, is present under weak stability, while data seem to scale better in the strongly stable regime.

In a first approximation, one may expect the results in Figure 4 to show an increasing deviation from the traditional scaling relations with increasing *complexity* of the underlying terrain where the data were measured (see the subjective ordering of the data sets in Table 1 and the associated colors). Therefore, it could be expected that the smallest discrepancies are found for the most ideal sites such as CASES‐99 (CA), Cabauw (CB), T‐RexC and i‐Box0 stations, and the biggest for data measured in stations located in very inclined terrain, such as i‐Box10 and i‐Box27 or i‐BoxTop. Figure 4, however, shows that this is not the case and there is little to no correlation between the deviation from traditional scaling and the a priori ordering of the data sets that was based solely on the slope angle‐induced complexity. In fact, it can be observed that the discrepancies in scaling are generally the largest for CASES‐99 and METCRAX II (Figures 4a–4c), despite these being some of the most a priori ideal locations (see Table 1). This mismatch with ordering of the data sets is present in all scaling variables, particularly in unstable and also in stable stratification, suggesting that our simple classification of data sets based on slope angle is not corresponding to the true nature of their complexity.

**Figure 5:**
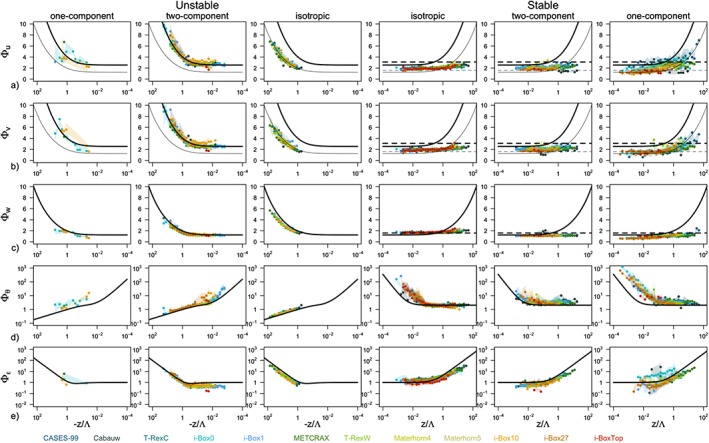
The same as Figure 3 but for data separated according to the pure states of anisotropy (isotropic, two‐component axisymmetric, and one‐component). The additional thin lines for the standard deviations of horizontal velocity components in (a) and (b) correspond to the scaling curve for Φ_w_.

**Figure 6:**
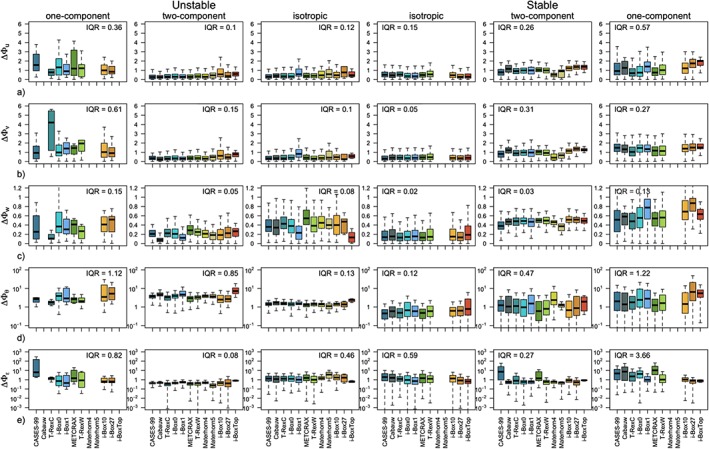
The same as Figure 4 but for turbulence separated according to the pure states of anisotropy. Note that here for isotropic turbulence Φ_w_ scaling relation is used as the reference.

Following the approach developed in Stiperski and Calaf (2018), next we separate the data according to the three limiting states of anisotropy: isotropic, two‐component axisymmetric and one‐component turbulence, before revisiting the scaling relations (Figure 5 ). Figure 6 shows the $\bar{\Delta \Phi_{y}}$ and *IQR* as objective measures of the improvement of the scaling. Mirroring the results for flat terrain (CASES‐99; Stiperski & Calaf, 2018), accounting for turbulence topology drastically improves scaling (i.e., decreases $\bar{\Delta \Phi_{y}}$ and *IQR*) for all data sets regardless of the complexity induced by terrain and local weather conditions. For all variables, the most consistent reduction in *IQR* and overall the best scaling behavior is apparent for isotropic turbulence both under unstable and stable stratification, and for unstable two‐component axisymmetric turbulence (Figure 6). Compared to Figure 4, the largest improvement is obtained for the horizontal velocity components under unstable stratification with $\bar{\Delta \Phi_{y}}$ and *IQR* reduced by up to 60%. As already found for CASES‐99 by Stiperski and Calaf (2018), isotropic and two‐component axisymmetric turbulence for horizontal velocity variances follow two distinctly different scaling lines. We come to the same conclusion as Stiperski and Calaf (2018) that this clustering of the data to two different scaling curves is the leading cause of the large scatter illustrated in Figures 3a, 3b, 4a, and 4b in the very unstable region. It is interesting to note that for all data sets, irrespective of complexity, isotropic turbulence occupies the same region of *z*/Λ as for flat terrain (i.e., *z*/Λ < −1). This is a clear sign of TKE production in isotropic turbulence being thermally dominated (Stiperski & Calaf, 2018) irrespective of terrain complexity. On the other hand, in complex terrain, two‐component axisymmetric turbulence is found also in very unstable conditions, contrary to the results over flat terrain (CASES‐99; Stiperski & Calaf, 2018). The small amount of data points corresponding to unstable one‐component turbulence does not allow us to reach definite conclusions about similarity of this type of turbulence. The results do seem to suggest a larger degree of scatter and therefore a lack of proper scaling.

In the stable regime, the scaled standard deviations of all velocity components follow the z‐less scaling up to *z*/Λ≈1 whereupon the data start to deviate from their constant value. This is opposite to the general expectations for z‐less scaling and might be an indication of self‐correlation (Klipp & Mahrt, 2004). The least scatter between the data sets (smallest *IQR*) is observed for isotropic turbulence. This is true even for the standard deviation of temperature, but only over a very limited range of stability. The near‐neutrally stable region of Φ_*θ*_, found by Sfyri et al. (2018) to be highly self‐correlated, shows large scatter. In addition, the negative slope of the Φ_*θ*_ scaling for isotropic and one‐component turbulence only appears at large stability than suggested by the curve for horizontally homogeneous and flat terrain. This *shifted* linear decrease in the relation between temperature variability and heat flux explains the comparatively large values of ΔΦ_*θ*_ (Figure 4d), that is, large deviations of the data sets from the scaling relations. The two‐component axisymmetric and one‐component turbulence show progressively more scatter (larger *IQR*) compared to isotropic turbulence but also a reduction of the value of the scaled standard deviations of velocity components (Φ_*u*,*v*,*w*_) and TKE dissipation rate (Φ_*ε*_), and an increase in the scaled standard deviation of temperature (Φ_*θ*_). This suggests that, at least for stable stratification, that is, when mechanically produced turbulence is being damped by negative buoyancy, anisotropic turbulence tends to have a larger temperature variance but smaller velocity variances in relation to the respective fluxes. One‐component turbulence is again the turbulence topology that exhibits most scatter, particularly for Φ_*u*_ and Φ_*ε*_. It is particularly interesting to note that the station with the most complex conditions (iBoxTop) shows largest deviations from scaling for unstable stratification but, on the other hand, does not exhibit systematic deviations for stable stratification but rather falls within those of the other data sets.

These results confirm our initial hypothesis that anisotropy is the key variable missing from scaling relations. Indeed, anisotropy seems to provide a direction toward a unifying framework for turbulence in conditions where the assumptions of Monin‐Obukhov Similarity Theory are generally violated, such as complex terrain or other sources of complex weather patterns that might affect the local flow.

**Figure 7:**
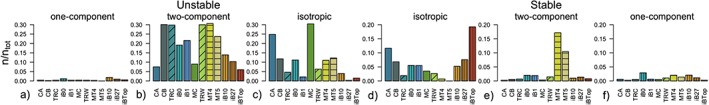
Frequency of occurrence (n/n_tot_) of pure states of anisotropy (one‐component, two‐component axisymmetric, and isotropic) for (a)–(c) unstable and (d)–(f) stable stratification. Here n is the number of averaging periods that are unstable/stable and at the same time belong to one of the pure anisotropy states, while n_tot_ is the total number of periods that are unstable/stable.

Because separating the data according to anisotropy does significantly improve scaling, we first attempt to explain the large scatter between and within the original data sets observed in Figures 3 and 4 by focusing on the frequency of occurrence of a given pure state of anisotropy. This is done to examine whether local topographic dissimilarities between the locations where the data sets were taken, cause different types of turbulence topologies to occur more or less frequently and converge toward different scaling curves, thus leading to large scatter if examined together. Figure 7 shows the number of averaging periods for each pure turbulence state *n* (separated according to anisotropy and stratification) divided by the total number of averaging periods that are unstable or stable *n*_tot_. One can first note that turbulence states classified within the *purely* isotropic, two‐ and one‐component regimes (cf. Figure 2) only represent a small fraction of the overall turbulence states. On average, the pure states of anisotropy jointly occur less than 40% of the time for unstable stratification and less than 10% for stable stratification. This means that only a smaller part of the data originally shown in Figure 3 fulfills the more restrictive criterion for the pure states of anisotropy (cf. Figure 5). However, it also illustrates that the more pure states of anisotropy are those that have a stronger impact on similarity scaling, attracting the data toward different scaling curves as seen in Figure 5. For example, both CASES‐99 and METCRAX II have the largest proportion of isotropic turbulence, which accounts for the largest scatter in scaled horizontal velocities in Figures 3 and 4 as mentioned above.

Stiperski and Calaf (2018) already showed that unstable isotropic turbulence occurs mostly under conditions of free convection away from the surface, when shear generation of turbulence is negligible (cf., convective regime of Kader & Yaglom, 1990). This coincides with the fact that all stations with prevailing isotropic turbulence are indeed located in areas that can be expected to frequently experience conditions supportive of free convection and generally have taller towers (flat terrain and more desert‐like location, e.g., CASES‐99, METCRAX II, MATERHORN, and i‐Box0). On the contrary, in complex terrain and close to the surface, isotropic turbulence hardly ever occurs (e.g., i‐Box27). The reason for this is the fact that in complex terrain thermally driven flows (slope and valley circulations), characterized by strong horizontal and vertical shear generation of turbulence (cf. Goger et al., 2018), develop in conditions that in flat terrain would support free convection (i.e., weak shear).

Given that different data sets were measured not only over different surfaces but also in different weather conditions, we cannot isolate the influence of terrain on the frequency of pure anisotropic states by examining all the data sets together. Therefore, we focus next only on data from data sets obtained from multiple towers in close proximity to each other and therefore experiencing similar weather conditions (e.g., T‐Rex, MATERHORN, and i‐Box). For example, T‐Rex Central and West tower appear to have almost identical percentages of pure states, thus suggesting that the slope angle does not play a major role on the anisotropy type there, at least not in unstable conditions. In stable conditions, the West tower on the slope has a marginally higher prevalence of isotropic data than the Central tower on the valley floor, suggestive of more developed turbulence there. Interesting are also the two MATERHORN towers both experiencing katabatic winds during nighttime (cf. Grachev et al., 2016), however, MT4 has a larger incidence of pure states than the MT5 tower, possibly due to its location in a less constrained topographic surrounding (open slope). For i‐Box sites, the frequency of unstable two‐component axisymmetric turbulence (stable isotropic turbulence) appears to decrease (increase) with increasing terrain complexity.

Still, even the classification according to the frequency of occurrence of pure states fails to identify patterns that connect anisotropy and turbulence complexity. The available methodology consequently appears to be inadequate to correctly describe the complexity of turbulence caused by both the terrain complexity (slope angle, heterogeneity, and land use) and complexity of the flow conditions. Therefore, we propose instead to use the deviation from traditional similarity scaling relations ( $\bar{\Delta \Phi_{y}}$) as a measure of complexity of a given data set. This measure incorporates the information on the interaction between terrain and flow complexity in forming the observed turbulence structure, and as such measures the departure from the canonical surface layer structure. The encouraging results presented above make us confident that anisotropy is the dominant process causing the departure from scaling for unstable stratification and that including information on it would improve scaling relations.We therefore hypothesize that these deviations from scaling exist due to the fact that scaling relations are evaluated in the physical, streamwise Cartesian coordinate system, whereas anisotropy is defined in the eigenvector reference frame. Similarly, Klipp (2018) suggested calculating the friction velocity in the eigenvector space as a means of improving scaling relations. Here we apply a different approach and instead use anisotropy as one of the explanatory variables that causes deviations from scaling curves. For stable stratification where anisotropy fails to improve scaling for two‐ and one‐component turbulence, we identify other physical mechanisms that could be responsible for the existence of complexity. Such an objective measure of complexity not only allows better comparison between data sets but it can provide a pathway for developing new universal scaling relations.

## 4 Quantifying Complexity

**Figure 8:**
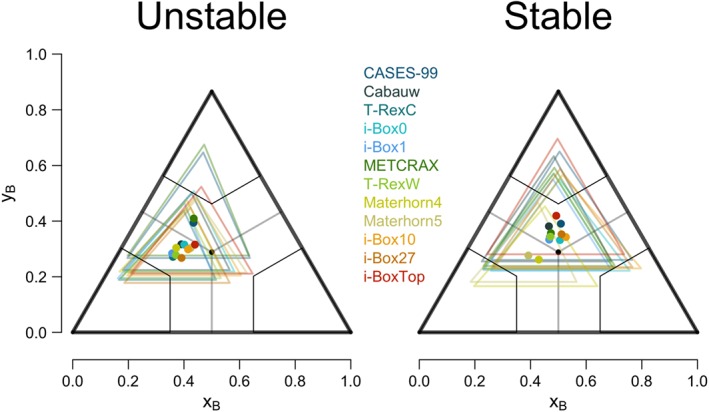
Barycentric Lumley Triangle showing the center of mass for all the data points within the triangle for each data set and stability. Different data sets are shown in color. Colored triangles represent the amount of spread of the data (calculated from the 75th percentile and are calculated around the center of mass).

We now take a step back and instead of focusing only on the pure states of anisotropy, we look at all the turbulence states (including mixed states) of anisotropy together. Figure 8 shows where the center of mass in the BLT resides for each data set. The size of the colored triangle represents the spread of the data and is calculated as the 75th percentile in x and y directions. The centers of mass show that, for unstable stratification, data are mostly centered between the isotropic and two‐component axisymmetric states, whereas for stable conditions they are more evenly spread between the two‐ and one‐component states but generally closer to the isotropic limit. The same as with frequency of occurrence of pure states, the information on the center of mass does not provide a conclusive information on the causes of turbulence complexity.

Complexity of turbulence in the atmospheric boundary layer can be caused by a number of processes acting on a range of scales. While we use the departure from the scaling curve as a measure of complexity, the causes of this departure have to be identified manually from a number of possible processes known to be relevant in complex terrain (cf. Serafin et al., 2018). These include (but are not limited to) the influence of terrain, where the easiest but not the only measure of terrain influence is the slope angle α. Although in the analysis so far, slope angle did not show a systematic influence on scaling, the inclination of terrain can still act indirectly, and this influence is therefore examined. Heterogeneity, although a significant source of complexity due to the formation of internal boundary layers as well as secondary circulations, is hard to quantify from experimental data and is therefore not examined here. Secondly, given the success of anisotropy in improving scaling in the results of the previous section, we examine anisotropy as a dominant variable influencing complexity. We use the coordinates of the BLT as scalar measures of anisotropy that encompasses all types of anisotropy (cf. Figure 8). Here y_B_ represents the shortest distance to pure isotropy similarly to what was used in Brugger et al. (2018), while x_B_ shows where in between two‐component axisymmetric and one‐component state the turbulence is situated. Two mesoscale processes are ubiquitous in complex terrain: thermally driven flows (upvalley/downvalley, upslope/downslope) and shallow water effects such as gravity waves. Thermally driven flows are characterized by significant wind turning with height (Rotach et al., 2008). The impact of this directional shear can be measured through the angle between the streamwise $\bar{u^{\prime }w^{\prime }}$ and spanwise $\bar{v^{\prime }w^{\prime }}$ momentum flux components, defined as

(17) $$\alpha_{vw}=\tan^{-1}(\frac{\bar{v^{\prime }w^{\prime }}}{\bar{u^{\prime }w^{\prime }}}).$$

If there is no directional wind shear with height, $\bar{v^{\prime }w^{\prime }}=0$, then α_vw_ will also be zero as all the vertical exchange of horizontal momentum will occur along the streamwise direction (recall that the double rotation orientates the coordinate system into the direction of the mean wind speed). The effect of wind turning on turbulence is therefore indirect, since it does not depend on driving parameters at the level where the momentum flux is measured but at heights below and above. This measure is convenient since it provides information on wind turning even if measurements are available only at one measurement level. Shallow water modes, such as gravity waves, may also affect turbulence (Sun et al., 2015) and can be quantified through the Froude number

(18) $$F_{r}=\frac{U}{\sqrt{gH}},$$

where H is the layer depth. Given that we have no way of determining the depth scale H from the available measurements, it has to be parameterized. For this purpose we use a modified boundary layer height following Zilitinkevich et al. (2012),

(19) $$H=\frac{\bar{w^{\prime }w^{\prime }}}{\sqrt{\left|f\bar{w\theta }g/\theta \right|}},$$

where f is the Coriolis parameter, and we use the vertical velocity variance instead of friction velocity, following Monti et al. (2002).

Finally, the influence of the smaller‐scale anisotropy, that is, anisotropy in the inertial subrange (cf. Katul et al., 1995; Poggi et al., 2003; Toschi et al., 2000), can be estimated through the ratio of turbulence dissipation rates

(20) $$\varepsilon_{vu}=\varepsilon_{v}/\varepsilon_{u},\varepsilon_{wu}=\varepsilon_{w}/\varepsilon_{u}.$$

Here ε_i_ is the dissipation rate as determined from the spectral density in the inertial subrange of the velocity component i. Babić and Rotach (2018) have shown that over heterogeneous surfaces these ratios, particularly ε_wu_, can deviate strongly from one.

**Figure 9:**
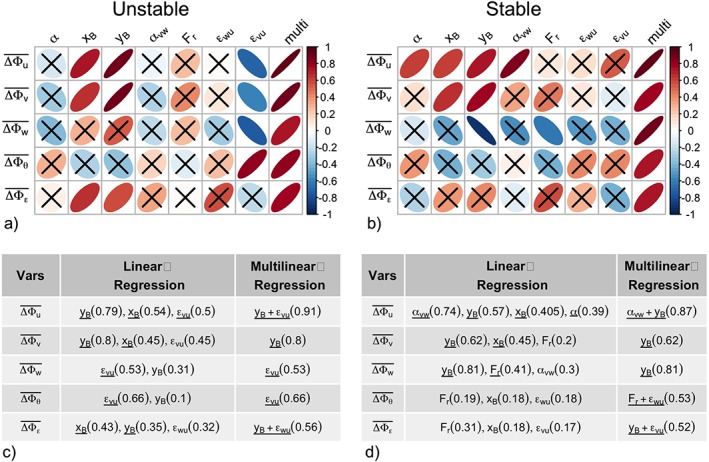
(a, b) Correlation matrix between the observed deviations from scaling ( $\bar{\Delta \Phi_{y}},y=u,v,w,\theta ,\varepsilon$) and the relevant variables representing physical processes for (a) unstable and (b) stable stratification. The correlation coefficients are shown in color and shape, so that perfectly circular shape means zero correlation and perfect line has correlation coefficient equal to 1. Crosses signify correlations that are not statistically significant at a 5% level. (c, d) List of statistically significant (p < 0.05) variables and their respective R ^2^ values from the linear and multilinear regression with $\bar{\Delta \Phi_{y}},y=u,v,w,\theta ,\varepsilon$ for (c) unstable and (d) stable stratification. Variables that are statistically significant are shown in bold.

**Figure 10:**
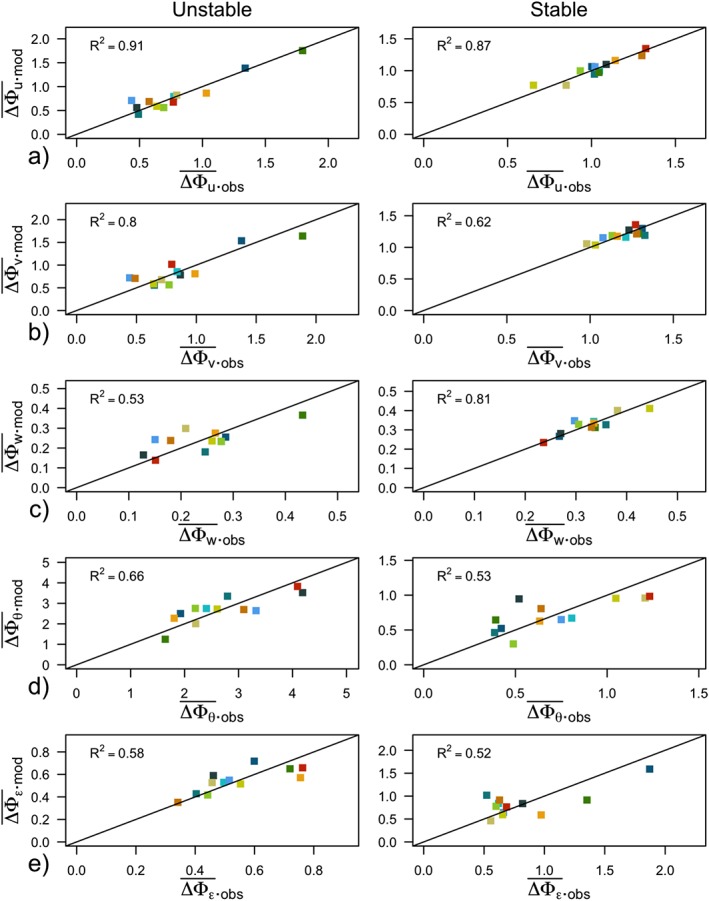
Comparison of observed deviations from scaling ( ${\bar{\Delta \Phi_{y}}}_{\text{obs}}$) and those modeled by multilinear regression ( ${\bar{\Delta \Phi_{y}}}_{\text{mod}}$) using variables from Figures 9c and 9d (column multilinear regression). Here y = u,v,w,θ,ε.

In order to identify which of these processes are relevant in complex terrain and therefore causing largest departures from scaling, we first individually employ the linear regression approach to determine the correlation between the departure from scaling $\bar{\Delta \Phi_{y}},y=u,v,w,\theta ,\varepsilon$ and the corresponding predictor variables (α,x_B_,y_B_,α_vw_,F_r_,ε_wu_,ε_vu_). The correlation coefficients are shown in Figure 9 for unstable and stable stratification. Second, a multlinear regression with the relevant variables was performed to estimate the joint influence of these processes on the departure from scaling. How many and which variables were chosen for the multilinear regression was determined in a step‐wise manner. From the step‐wise procedure we choose as final the combination of statistically significant variables (p < 0.05) with the largest R ^2^ and smallest Bayesian Information Criterion (BIC; Wilks, 2011). The variables in linear and multilinear regression are shown in Figures 9c and 9d, and the observed departures from scaling and the ones predicted by multilinear regression are shown in Figure 10. The largest limitation of this approach is of course the assumption of a linear relationship between predictors and the explanatory variable.

The results of both linear and multilinear regression (Figure 9) show that *y*_*B*_ and therefore the shortest distance to isotropic state is truly one of the most important explanatory variables both in unstable and stable stratification, confirming the results of the previous section. Indeed, *y*_*B*_ can explain up to 80% of variability between the complex terrain scaling relations for certain variables and stability ranges. The correlation with *y*_*B*_ is positive, indicating that the increase of complexity coincides with the departure from isotropic conditions. In the unstable regime the other important process influencing complexity is the small‐scale turbulence anisotropy shown through *ε*_*vu*_. Although the value of this ratio is within the 20% margin of one and therefore could be considered almost constant (not shown), the individual values show a negative correlation with increasing complexity (apart from scaled temperature), indicating interestingly that the turbulence with a higher degree of horizontal small‐scale isotropy is found in more complex conditions (cf., anisotropy above canopy, Brugger et al., 2018). These results are also contrary to Babić and Rotach (2018) where *ε*_*wu*_ diverges more from one. In the stable regime where anisotropy was successful in improving scaling only in isotropic conditions, the mesoscale processes appear to be more important than in the unstable regime. Therefore, wind turning with height (*α*_*vw*_) and to a lesser degree the Froude number *F*_*r*_ appear to additionally explain an important part of the observed complexity.

The clear connection between ΔΦ_*u*,*v*_ and *y*_*B*_ comes as no surprise given previous evidence that isotropic and two‐component turbulence occupy different scaling curves, so that the distance to isotropy clearly delineates stations that have different percentages of these pure states and therefore cluster around them. On the other hand, in stable stratification, it is the deviation of vertical velocity variance ΔΦ_*w*_ that (anti)correlates best with *y*_*B*_ suggesting that unlike in unstable stratification, it is in the vertical velocity component that anisotropy shows largest differences. This is intuitive given that as stratification increases, the vertical velocity variance decreases from isotropic toward one component with a progressively lower neutral limit, as observed in Figure 5. The importance of distinguishing between the very anisotropic states (two‐ and one‐component, i.e., *x*_*B*_) is also clearly identified as important for stable stratification, where one‐component turbulence occurs more frequently. The existence of wind turning with height appears to have the largest influence on ΔΦ_*u*_ in stable conditions, whereas scalar variances appear to be most affected by gravity waves. The fact that the results are dependent on the ratio of the dissipation rates points to the scale‐dependence of anisotropy and the persistence of anisotropy to very small scales. Toschi et al. (2000) have shown that this might be due to the effect of wind shear persisting across all scales.

Figure 9 shows that the multlinear combination of the above identified processes explains the majority of the variance for the standard deviation of velocity components, and to a lesser degree of the scalar variances (temperature and TKE dissipation rate) indicating the dissimilarity between the momentum and scalars (Brutsaert, 1982), but also pointing toward missing processes that have either not been identified by our limited list or are nonlinear and therefore are not detected by the linear method.

## 5 Discussion and Conclusions

The results of the previous sections have highlighted the importance of anisotropy in shaping the scaling relations, and have therefore shown that the approach of Stiperski and Calaf (2018) accounting for anisotropy, significantly improves scaling even over highly complex terrain. The large site‐to‐site variability in turbulence structure commonly found over complex terrain was then shown to be due to the differences in the frequency of occurrence of each anisotropy type, causing large scatter in the data, as different anisotropy states follow different scaling curves. For unstable stratification, anisotropy was found to be the dominant processes causing the failure of scaling among the various data sets. Whether the isotropic state or the two‐component turbulence should actually be taken as the reference state in unstable stratification remains a question—especially since the atmospheric boundary layer turbulence is determined by the interplay between shear‐ and buoyancy‐dominated turbulence (and the former is by definition anisotropic). Given the fact that two‐component turbulence is more prevalent in unstable stratification and the data fit classic scaling relations better, suggests that the two‐component limit is the reference state for unstable stratification. For stable stratification, isotropic turbulence is clearly the reference turbulence state, corresponding to weakly stable boundary layers with well‐developed turbulence (cf. Stiperski & Calaf, 2018). In stable stratification, however, we see that anisotropy itself cannot explain all the variability observed. It was therefore shown that physical mechanisms, such as directional wind shear, as well as effects of mean wind speed gradients persisting to the smallest scales and affecting turbulence in the inertial subrange, cause the complexity of turbulence. While these processes obviously occur also over flat and homogeneous terrain, they are more frequent and their effects more pronounced in complex terrain.

**Figure 11:**
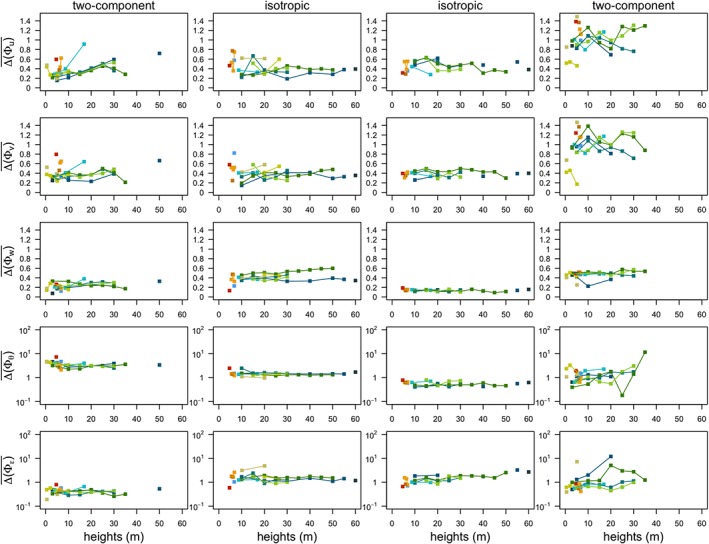
Median absolute deviations from scaling $\bar{\Delta \Phi_{y}},y=u,v,w,\theta ,\varepsilon$ as function of measurement height for unstable two‐component axisymmetric and isotropic turbulence and stable isotropic and two‐component axisymmetric turbulence.

Another relevant issue associated with turbulence in complex terrain is the depth of the boundary layer (De Wekker & Kossmann, 2015; Lehner & Rotach, 2018; Rotach & Zardi, 2007), and consequently of the surface layer. Although we are employing local scaling (and not the Monin‐Obukhov similarity scaling) and therefore do not require that the measurement levels be strictly within the surface layer, it is important to investigate the validity of this hypothesis. We therefore examine the median absolute deviations from scaling ( $\bar{\Delta \Phi_{y}}$) as functions of measurement height for data separated according to anisotropy (Figure 11). Comparing the influence of height (stability) on scaling, shows that the height dependence within a given tower is on the same order as the site to site variability. Indeed, the deviations from scaling show almost no height dependence for isotropic turbulence, particularly for Φ_*w*_,Φ_*θ*_, and Φ_*ε*_. There is a larger but nonsystematic variability for the horizontal velocity components under conditions of two‐component axisymmetric turbulence, particularly for stable stratification, suggestive of intermittent conditions and layering associated with this type of very stable turbulence.

These results suggest that the physics represented by the newly introduced variables *y*_*B*_, *ε*_*vu*_, *α*_*vw*_, and *F*_*r*_ should be considered when working on the development of new scaling relations. Additionally, the present results highlight the possibility of using multilinear regression expressions, such as found in this work, as a more effective way of determining complexity of a given data set than traditional measures, such as slope angle. It is quite likely, however, that at least part of the remaining scatter between the data and the prediction of multilinear regression (cf., Figure 9) is associated with spatial (horizontal) heterogeneity, which is inherent in complex terrain but difficult to assess from single‐tower observations. Before expressions accounting for complexity in scaling relations can be used, this study would have to be extended to more than 12 data sets to improve its statistical significance. Of particular need in this respect would be horizontally distributed and long‐term turbulence measurements from tall towers over very complex mountainous terrain. Despite the current approach being diagnostic, it is already clear that this methodology provides a direction toward a universal theory of near‐surface turbulence in terrain of all kinds of complexity. This will have particularly large implications for numerical modeling of weather and climate, where turbulence is parameterized by using scaling relations developed and hence only valid over flat and horizontally homogeneous terrain.

## Supporting information



Supporting Information S1
