# Supplementary material for: Scaling, Anisotropy, and Complexity in Near‐Surface Atmospheric Turbulence
Source: J Geophys Res Atmos. 2019 Feb 8;124(3):1428–48. doi: 10.1029/2018JD029383 (PMC6392143; doi:10.1029/2018JD029383)
Supplement: Supplementary file 1 — Supporting Information S1 [file JGRD-124-1428-s001.pdf]

AGU PUBLICATIONS

Journal Name of Geophysical Research Atmosphere

Supporting Information for

Scaling, anisotropy and complexity in near-surface atmospheric turbulence

Ivana Stiperski[1], Marc Calaf[2], Mathias W. Rotach[1]

[1]Department of Atmospheric and Cryospheric Sciences, University of Innsbruck, Innsbruck, Austria
[1]Department of Mechanical Engineering, University of Utah, Salt Lake City, Utah, USA

Additional Supporting Information (Files uploaded separately)

ScalingAnisotropyComplexity_data.zip

Introduction

This supporting information provides the data from the twelve datasets used in this study. The files are compressed in the file ScalingAnisotropyComplexity_data.zip consisting of twelve files in the csv format, named ScalingAnisotropyComplexity_Datasetname.csv, where Datasetname follows the naming defined in Table 1 of the paper (CASES-99, Cabauw, i-Box0, i-Box1, i-Box10, i-Box27, i-BoxTop, MATERHORN ES4, MATERHORN ES5, METCRAX II, T-RexC and T-RexW).

Each data file consists of columns of vectors for each variable (see variable names below). Data are not ordered according to the date but according to the height and stability: unstable data for a given height are listed first and consist of 30-min averages, followed by 1-min-average stable data. The data are quality-controlled, stationarity and the Richardson number criteria has been applied.

List of variable names and description:
ZoL - "z/L" - Local stability where L is the local Obukhov length
SigmaU - "sigmaU/u*" - Local scaled standard deviation of stream-wise velocity (u)
SigmaV - "sigmaV/u*" - Local scaled standard deviation of span-wise velocity (v)

SigmaW	- "sigmaW/u*"	- Local scaled standard deviation of surface-normal velocity (w)
SigmaT	- "sigmaT/T*"	- Local scaled standard deviation of temperature
SigmaEU	- Local scaled TKE dissipation rate of stream-wise velocity (u)	
SigmaEV	- Local scaled TKE dissipation rate of stream-wise velocity (v)	
SigmaEW	- Local scaled TKE dissipation rate of stream-wise velocity (w)	
height	- Measurement height [m]	
xB	- x-coordinate (invariant) of the Lumley Barycentric Map	
yB	- y-coordinate (invariant) of the Lumley Barycentric Map	
uw	- stream-wise momentum flux [m^2 s^{-2}]	
vw	- span-wise momentum flux [m^2 s^{-2}]	
Fr	- Froude number	
